# Global spatiotemporal biomechanics using video swin transformer: multiscale validation and clinical impact for keratoconus suspects

**DOI:** 10.1038/s41746-026-02751-x

**Published:** 2026-05-16

**Authors:** Xuan Chen, Zuoping Tan, Yimei Han, Tinghui Huang, Rui Yao, Qinghong Gao, Shuangcheng Li, Jing Li, Shu’an Liu, Caiye Fan, Guoxing Zhao, Vishal Jhanji, Yuanyuan Wang, Yan Wang

**Affiliations:** 1 School of Medicine, Nankai University Eye Institute, Nankai University, Tianjin, China; 2 Tianjin Eye Hospital, Tianjin Key Lab of Ophthalmology and Visual Science, Tianjin Eye Institute, Nankai University Affiliated Eye Hospital, Tianjin, China; 3 Wenzhou University of Technology, Wenzhou, Zhejiang China; 4 Clinical College of Ophthalmology, Tianjin Medical University, Tianjin, China; 5 Shaanxi Eye Hospital, Xi’an People’s Hospital, Xi’an, Shaanxi China; 6 Department of Ophthalmology, University of Pittsburgh School of Medicine, Pittsburgh, PA USA

**Keywords:** Diseases, Engineering, Health care, Medical research

## Abstract

Biomechanical assessment offers a novel approach for the diagnosis and treatment of corneal ectatic disorders, particularly keratoconus, a blinding eye disease characterized by biomechanical weakening. However, current early diagnosis primarily relies on morphology, such as corneal tomography, which can lead to missed cases. This study utilized a video swin transformer to characterize global spatiotemporal corneal biomechanics from deformation videos. This model captures the entire dynamic process, significantly enhancing the diagnostic accuracy for suspicious cases (AUC 0.9942; accuracy 97.37%; 95% CI: 93.0%, 99.7%). Temporal-importance analysis revealed rebound-phase energy dissipation as a key early marker. Additionally, atomic force microscopy-based nanoindentation and adhesion tests confirmed reduced apparent Young’s modulus and increased adhesion force in keratoconus. In conclusion, this study presents a novel, interpretable, in vivo, video-driven framework that supports early differential diagnosis and personalized intervention. This approach has clinical potential for precision medicine.

## Introduction

The cornea, a crucial optical tissue in the eye, plays a vital role in maintaining morphological stability, refractive status, and responding to mechanical loads like intraocular pressure^1^. Under normal conditions, the cornea exhibits remarkable biomechanical properties, allowing it to deform under external forces and rapidly return to its original shape upon removal. However, certain ophthalmic diseases, such as keratoconus (KC) and glaucoma, disrupt these biomechanical responses, leading to abnormal deformations. KC is a progressive blinding eye disease characterized by progressive corneal thinning and protrusion, driven by a positive feedback loop of biomechanical decompensation and tissue remodeling^2^. Conventional examinations like slit-lamp microscopy and corneal tomography often fail to reliably differentiate and diagnose keratoconus suspects (KCS) due to the unclear nature of early-stage manifestations. In contrast, corneal biomechanical changes are believed to occur earlier and are more sensitive to subtle lesions^3,4^. Consequently, early diagnosis and treatment strategies targeting biomechanics have become increasingly important in ophthalmology and visual science.

As early as two decades ago, corneal biomechanics research initially focused on ex vivo testing^5^. The classic ex vivo tensile test reveals that KC corneas sustain lower stress under the same strain, indicating that the cornea becomes softer^6^. Notably, AFM studies have revealed that corneal stromal biomechanics exhibit depth-dependent properties and spatial heterogeneity, indicating that KC-associated mechanical abnormalities cannot be simplified as a global, uniform reduction in elasticity^7–10^. Multimodal imaging methods, such as X-ray scattering, polarization/phase second-harmonic generation (SHG), have further shown elevated disorganization in collagen lamellar orientation and decreased crosslinking in KC. These microstructural abnormalities spatially coincide with the distribution of focal softening^11–13^. With the advancement of precision medicine, a key challenge is to transform the accumulated foundational and mechanistic insights into crucial assessment techniques that are closely associated with the pathophysiological process, and for detecting KC during the early stage. Addressing this challenge is crucial for decreasing the risk of corneal transplantation and ultimately protecting visual health.

In recent years, in vivo corneal biomechanical evaluation methods have rapidly developed. For instance, an ocular response analyzer (ORA) measures corneal deformation curves using noncontact optical reflection to assess viscoelastic properties^14^. The Corneal Visualization Scheimpflug Technology (Corvis ST) uses high-speed Scheimpflug imaging technology to record the dynamic corneal deformation process under an air puff and derives a series of biomechanics-related parameters^15^. The Brillouin microscope is a non-invasive, light-scattering–based method that measures the corneal longitudinal modulus^16^. Optical coherence elastography (OCE) combines optical coherence tomography (OCT) with elastography methods to quantitatively examine tissue biomechanics with high spatial resolution^17^. Overall, most existing devices remain at the static, single, and overall parameter level, and have not yet achieved comprehensive and region-specific characterization of the corneal force–deformation processes.

Analytical techniques in corneal biomechanics have evolved from traditional statistics to machine learning (ML) and, more recently, deep learning (DL). Classic algorithms, such as support vector machine (SVM), random forest (RF), and XGBoost, have been previously investigated to enhance the identification of KC^18–20^. Ambrósio and Vinciguerra et al. developed multiparameter models, improving early KC detection^21,22^. Previously, our group used a feedforward neural network combined with key biomechanical features and achieved a diagnostic accuracy of nearly 100% for KC^23^. The diagnostic accuracy of early KC was also substantially enhanced by characterizing localized corneal biomechanics with pixel-level parameters and utilizing an ensemble learning model^24^. Then proposed an unsupervised shared model that facilitates high-precision contour extraction, achieving more accurate and reliable biomechanical assessment^25^. Recently, Lombardo et al. extracted deformation profiles from Corvis videos and fitted them to viscoelastic models to estimate corneal stiffness-related parameters, thereby providing a valuable quantitative framework for corneal biomechanics studies^26,27^. Nevertheless, the analysis of corneal biomechanical videos remains at an early stage overall^28^, making it challenging for clinicians to directly identify the core factors underlying lesion development. Therefore, this limits the clinical interpretability and usability.

To overcome this limitation, we propose an intelligent analytical approach that can directly analyze the sequence of videos and track the spatiotemporal dynamics of corneal deformation. Recently, transformer-based models have demonstrated superior performance in medical video analysis and have been successfully applied to tasks such as ultrasound video processing, endoscopic video segmentation, and neuroimaging analysis^29,30^. Building on these advancements, we present a video swin transformer (VST)-based framework for global dynamic biomechanical characterization. Furthermore, we applied techniques such as Gradient-weighted Class Activation Mapping (Grad-CAM) to quantify the contributions of various time points and spatial regions to the diagnostic prediction, thereby revealing key nodes in the evolution of corneal biomechanics. Collectively, this framework is expected to assist clinicians in identifying potential KCS earlier and more accurately, thereby enhancing diagnostic sensitivity and improving the precision of treatment decision-making.

## Results

### Baseline characteristics of the participants and dataset partitioning

This study included 768 corneal dynamic deformation videos of 768 eyes (768 participants). The dataset was divided into training, test, and validation sets at ratios of 70%, 15%, and 15%, respectively. The proportions of the three subject groups remained constant and balanced across all subsets. Overall, 85% of the samples (*n* = 654), comprising 218 NC, 218 FFKC, and 218 KC, were utilized for model development (training + internal testing). And 15% of the samples (*n* = 114) were used as an independent external validation set to ensure comparable category distributions between the model development and evaluation phases.

The age distributions of the three groups were consistent across various dataset subsets. The mean ages of the NC group were 23.21 ± 5.98 years in the development cohort and 23.31 ± 5.16 years in the external validation cohort; those of the FFKC group were 22.33 ± 5.58 years and 23.14 ± 6.27 years, respectively; and those of the KC group were 22.54 ± 5.36 years and 23.04 ± 4.35 years, respectively. ANOVA revealed no statistically significant differences in the age distributions between the subsets within each group (all *P* > 0.05). Therefore, this reduced the potential effect of age-related confounding factors on model training and generalization evaluation.

Furthermore, we performed intergroup comparisons to clarify the potential confounding effects of IOP- and CCT-related factors. The results showed that IOP did not differ significantly among the groups (F = 1.124, *P* = 0.326), whereas CCT differed significantly (F = 35.009, *P* < 0.001). Baseline characteristics, including sample size, age, and sex, are summarized in Table 1.

**Table 1:** Participants’ baseline characteristics

	Sample dataset			Validation dataset			*P*-value
Characteristics	NC	FFKC	KC	NC	FFKC	KC	-
Sex (M/F)	133/85	160/58	141/77	23/15	25/13	22/16	-
Age	23.21 ± 5.98	22.33 ± 5.58	22.54 ± 5.36	23.31 ± 5.16	23.14 ± 6.27	23.04 ± 4.35	*P*^a^ = 0.383 *P*^b^ = 0.504 *P*^c^ = 0.297
K1 (D)	42.17 ± 1.72	42.49 ± 1.32	46.38 ± 3.82	42.16 ± 0.99	42.83 ± 1.37	45.45 ± 3.23	*P*^a^ = 0.748 *P*^b^ = 0.281 *P*^c^ = 0.131
K2 (D)	43.45 ± 1.94	43.96 ± 1.47	50.07 ± 4.83	43.34 ± 1.32	44.23 ± 1.39	48.84 ± 4.39	*P*^a^ = 0.789 *P*^b^ = 0.540 *P*^c^ = 0.102
Kmax (D)	44.02 ± 2.65	45.06 ± 1.74	56.36 ± 7.55	43.84 ± 1.44	45.52 ± 1.88	54.19 ± 6.27	*P*^a^ = 0.774 *P*^b^ = 0.276 *P*^c^ = 0.095
IOP (mmHg)	15.64 ± 2.33	14.96 ± 3.84	14.94 ± 2.42	15.09 ± 2.44	14.56 ± 3.43	14.93 ± 2.73	*P*^a^ = 0.846 *P*^b^ = 0.476 *P*^c^ = 0.108
CCT (μm)	558.99 ± 24.39	509.38 ± 34.25	472.07 ± 34.85	556.71 ± 21.81	512.18 ± 30.12	474.97 ± 41.95	*P*^a^ = 0.525 *P*^b^ = 0.667 *P*^c^ = 0.449
KI	1.03 ± 0.04	1.05 ± 0.04	1.21 ± 0.12	1.02 ± 0.02	1.05 ± 0.04	1.19 ± 0.09	*P*^a^ = 0.897 *P*^b^ = 0.386 *P*^c^ = 0.337
CKI	1.01 ± 0.02	1.01 ± 0.07	1.08 ± 0.06	1.00 ± 0.01	1.01 ± 0.01	1.06 ± 0.05	*P*^a^ = 0.132 *P*^b^ = 0.173 *P*^c^ = 0.081
BAD-D	0.83 ± 1.28	2.40 ± 1.07	8.70 ± 3.81	0.70 ± 0.50	2.43 ± 0.97	7.71 ± 3.48	*P*^a^ = 0.587 *P*^b^ = 0.724 *P*^c^ = 0.070
CBI	0.17 ± 0.16	0.42 ± 0.36	0.96 ± 0.11	0.18 ± 0.16	0.41 ± 0.35	0.95 ± 0.08	*P*^a^ = 0.822 *P*^b^ = 0.915 *P*^c^ = 0.105
TBI	0.12 ± 0.15	0.71 ± 0.34	0.99 ± 0.08	0.11 ± 0.13	0.72 ± 0.35	1.00 ± 0.00	*P*^a^ = 0.939 *P*^b^ = 0.725 *P*^c^ = 0.375

*BAD-D* Belin/Ambrósio Deviation, *CBI* Corvis Biomechanical Index, *CCT* center corneal thickness, *CKI* center keratoconus index, *D* diopters, *F* female, *IOP* intraocular pressure, *K1* flat keratometry of the corneal anterior surface, *K2* steep keratometry of the corneal anterior surface, *KC* keratoconus, *KI* keratoconus index, *Kmax* maximum keratometry, *M* male, *NC* normal corneas, *TBI* Tomographic and Biomechanical Index.

Differences between the sample and validation datasets were evaluated using ANOVA (P^a^: NC; P^b^: FFKC; P^c^: KC).

### Diagnostic performance of FFKC using global spatiotemporal biomechanical characterization extracted from full-length videos by VST

**Fig. 1:**
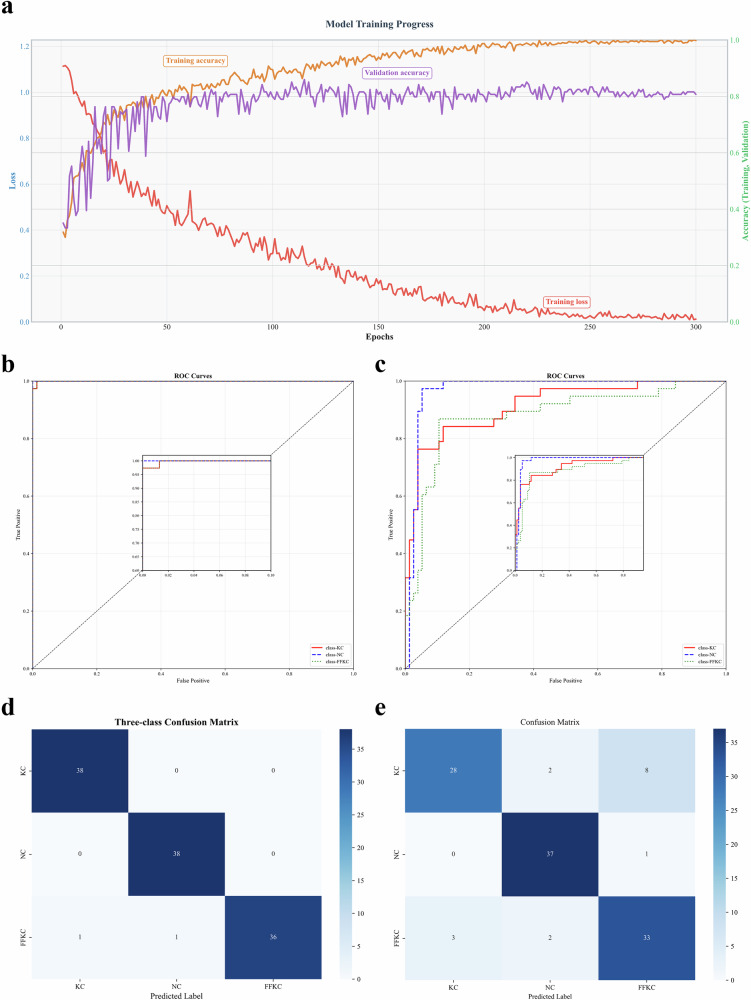
Model performance. **a** The training curves for the best-performing model; **b** The Receiver Operating Characteristic (ROC) curves on the Test Dataset; **c** The ROC curves on the Validation Dataset; **d** Confusion Matrix of the Model on the Test Dataset; **e** Confusion Matrix of the Model on the Validation Dataset.

After artifact suppression and temporal standardization, the entire corneal deformation video, which covered the full sequence from loading to maximum deformation and subsequent recovery, was fed end-to-end into a VST to automatically extract global spatiotemporal biomechanical characterizations from the complete deformation process. VST operates on 3D patches and uses multihead self-attention within local 3D windows to model coupled spatiotemporal features. A shifted-window mechanism further facilitates cross-window interactions between adjacent regions. This allows long-range dependencies and multi-scale information to be progressively integrated into a hierarchical feature pyramid. Unlike conventional methods, which rely only on a few static parameters, this architecture preserves temporal continuity while finely characterizing the dynamic differences across the applanation, maximum deformation, and rebound phases. Therefore, this provides more sensitive discriminative features for FFKC, which generally shows subtle and localized biomechanical abnormalities. Finally, the model performs video-level classification via global pooling followed by a classification head. During training, loss and accuracy steadily converged with the iterations (Fig. 1a).

In terms of the overall performance, the model achieved an accuracy of 97.37% (95% CI: 93.0–99.7%) on the test set and 95.61% (95 CI: approximately 90.5–97.8%) on the external validation set, suggesting that it maintained a good fit while showing a certain degree of cross-domain generalizability. Class-wise ROC analysis using a one-vs.-rest strategy revealed AUC values of nearly 1.0000 for all three categories (KC = 1.0000, FFKC = 0.9900, and NC = 0.9927; Fig. 1b, c), indicating robust discriminative capability across a wide range of decision thresholds.

In terms of the class-specific performance on the test set, the accuracy of FFKC was 92.11% (95% CI: 79.2–97.8%), with a 92.11% recall and a 95.89% F1 score. For KC, the accuracy reached 100% (95% CI: 90.4–100%), with a 100% recall and a 98.70% F1 score. For NC, the accuracy was 100% (95% CI: 90.4–100%), with a 100% recall and a 97.44% F1 score. Figure 1d shows the corresponding confusion matrix. In the external validation set, the accuracy of FFKC was 92.11% (95% CI: 79.2–97.8%), with a 92.11% recall and a 93.33% F1 score. KC achieved a 97.37% accuracy (95% CI: 86.9–99.6%) with a 97.37% recall and a 94.87% F1 score. Additionally, the NC achieved a 97.37% accuracy (95% CI: 86.9–99.6%), with a 97.37% recall and a 98.67% F1 score. Figure 1e shows the confusion matrix for the external validation. In addition, we performed five-fold cross-validation. The results showed that the model maintained strong and consistent performance on the external validation cohort, with a mean AUC of 99.54% ± 0.36% for KC, 98.04% ± 0.33% for NC, and 95.69% ± 0.82% for FFKC. These findings indicate good robustness and stability of the model. Collectively, these results indicate that the model maintains an extremely high sensitivity for the NC while providing a strong discriminative capability for differentiating FFKC from KC.

Compared with the commonly used composite clinical indices, the VST showed a particularly pronounced advantage in identifying FFKC. In the test and external validation sets, VST’s diagnostic accuracy for FFKC (92.11% and 92.11%, respectively) was significantly higher than that for BAD-D (88.05% and 86.80%, *P* < 0.01), CBI (70.75% and 68.45%, *P* < 0.01), and TBI (88.75% and 88.15%, *P* < 0.01) (Table 2). These results further support that spatiotemporal dynamic biomechanical characterization derived from full-length deformation videos can capture subtle dynamic differences that are challenging to quantify using traditional static parameters, thereby providing greater diagnostic gain and potential clinical value for the early FFKC detection.

**Table 2:** Diagnostic accuracy of BAD-D, CBI, and TBI for FFKC on the Test and Validation Datasets

	AUC	Cutoff	Accuracy	Sensitivity	Specificity	*P*-value
Test Dataset						
BAD-D	0.922	1.465	88.05%	80.20%	95.90%	<0.01
CBI	0.656	0.379	70.75%	47.90%	93.60%	<0.01
TBI	0.927	0.300	88.75%	86.20%	91.30%	<0.01
Validation Dataset						
BAD-D	0.904	1.64	86.80%	71.60%	84.00%	<0.01
CBI	0.655	0.241	68.45%	63.20%	73.70%	<0.01
TBI	0.906	0.249	88.15%	89.50%	86.80%	<0.01

*BAD-D* Belin/Ambrósio Deviation, *CBI* Corvis Biomechanical Index, *TBI* Tomographic and Biomechanical Index.

### Biomechanical progression trajectories and probability calibration with reliability analysis

**Fig. 2:**
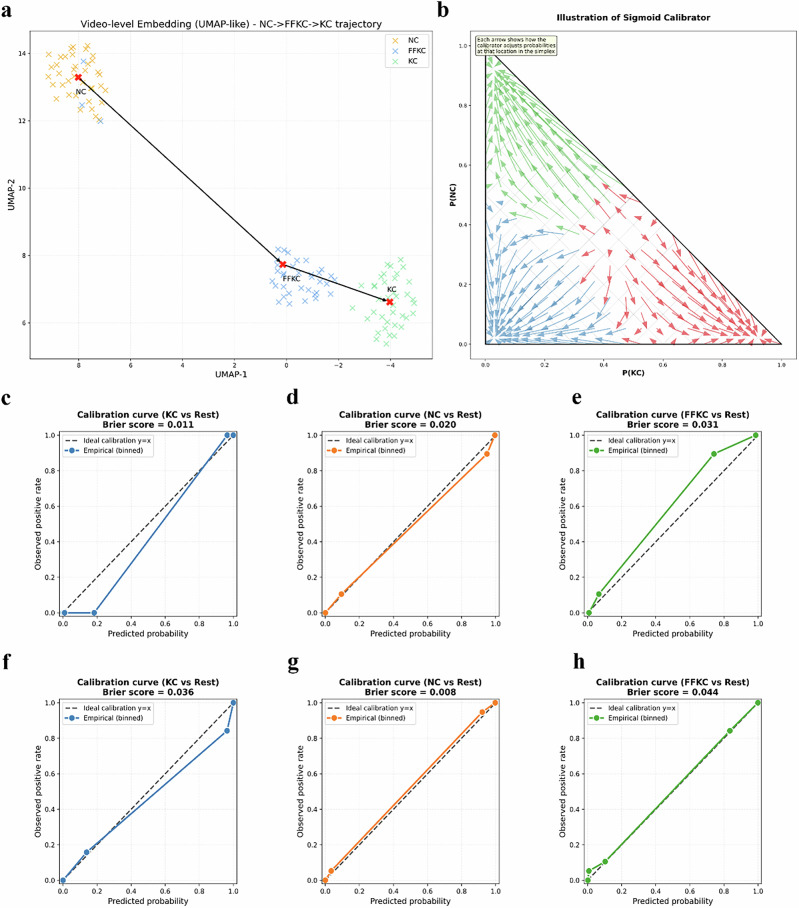
Biomechanical progression trajectories, probability calibration, and reliability analysis. **a** UMAP embedding illustrating the clustering of NC/FFKC/KC and the biomechanical progression trajectory along the NC-FFKC-KC axis; **b** Schematic illustration of the sigmoid calibrator in the three-class probability simplex, with arrows indicating the direction and magnitude of how the calibrator maps raw predicted probability vectors to calibrated probability vectors; **c**–**e** One-vs-rest calibration curves for the three classes in the test set; **f**–**h** One-vs-rest calibration curves for the three classes in the validation set.

We extracted video-level embedding vectors generated by the VST and applied UMAP to reduce them to a two-dimensional representation to examine the continuity and potential evolutionary direction of corneal biomechanical phenotypes across disease subtypes. Therefore, we constructed a biomechanical trajectory map. NC, FFKC, and KC were naturally separated into three relatively compact clusters in the embedding space, whereas the overall distribution showed a monotonic progression along an NC-FFKC-KC axis, spanning normal, subclinical, and overt KC (Fig. 2a). A small number of transitional samples were still observed near the inter-cluster boundaries. The FFKC cluster was geometrically positioned between NC and KC, further supporting its phenotypic characterization as a transition state or early subtype. This suggests that the model learned a continuous biomechanical gradient in the latent space, which is consistent with disease progression. Notably, these outputs may help clinicians understand what the model predicts as well as assess how close a specific eye is to a particular biomechanical state.

We calibrated the predicted probabilities and systematically evaluated the model’s reliability using internal and external datasets to enhance the clinical interpretability of the model’s probabilistic outputs. Figure 2b shows the role of the sigmoid calibrator on the three-class probability simplex, where each point within the simplex represents a three-class predicted probability vector and the three vertices correspond to complete confidence in a single class. The calibrator maps and redistributes the raw predictions in the probability space to correct for overconfidence or underconfidence, thereby making the predicted probabilities closer to the true event rates. In this simplex, the region near the upper-left corner corresponds to a high P (NC), the lower-right corner to a high P (KC), and the lower-left corner to a high P (FFKC). Therefore, this provides a more reliable probabilistic basis for threshold selection and risk stratification.

Furthermore, the reliability diagrams (calibration curves) plotted using a one-vs. rest strategy revealed that in the test set, the curves for all three classes closely followed the ideal diagonal line (Fig. 2c–e). The corresponding Brier scores were 0.011 for KC, 0.020 for NC, and 0.031 for FFKC, indicating a good calibration quality of the predicted probabilities on the internal test set. Notably, the Brier score for the FFKC was slightly higher, which is consistent with the expectation that this boundary and transitional class are more prone to probability miscalibration. The calibration for NC remained good (Brier = 0.008) in the external validation set, whereas the calibration errors increased for KC and FFKC (KC Brier = 0.036; FFKC Brier = 0.044; Fig. 2f–h). This suggests that distribution shifts across devices or populations may compromise the probabilistic outputs’ reliability, with FFKC being especially sensitive owing to its proximity to the decision boundary.

### Spatiotemporal interpretability and clinical consistency underscore rebound-phase energy dissipation as an additional key cue

**Fig. 3:**
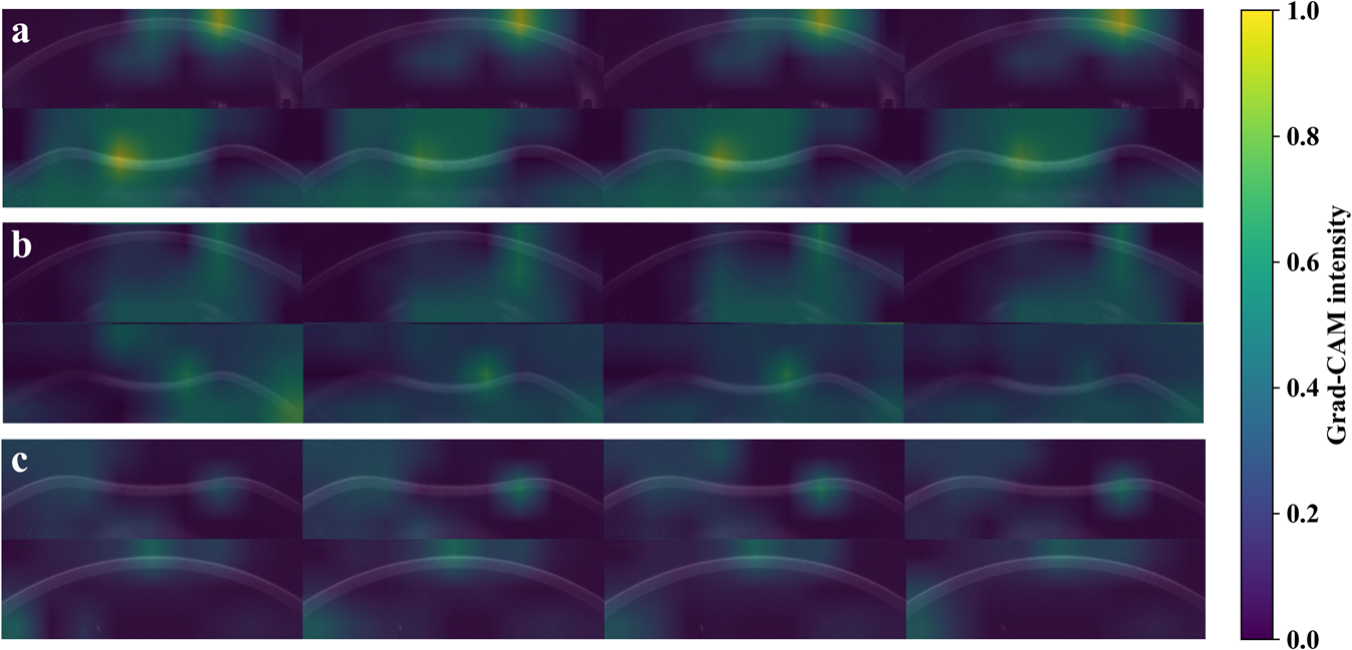
Heat map representation. **a** KC; **b** FFKC; **c** NC.

We performed a full-sequence interpretability analysis (Grad-CAM) on all videos after artifact suppression and temporal standardization to elucidate the advantages of VST in early FFKC identification, providing temporal attribution and spatial localization throughout the process of corneal applanation, maximum deformation, and rebound. Heatmaps overlaid on the key frames from representative cases (Fig. 3) revealed that the model consistently assigned close attention weights to the central or paracentral region before and after applanation and maintained this focus continuously over the dynamic sequence rather than fluctuating across isolated frames. This pattern is consistent with our end-to-end modeling strategy using full-length videos. This indicates that the model prioritizes the overall deformation process and its dynamics rather than solely relying on static morphology.

**Fig. 4:**
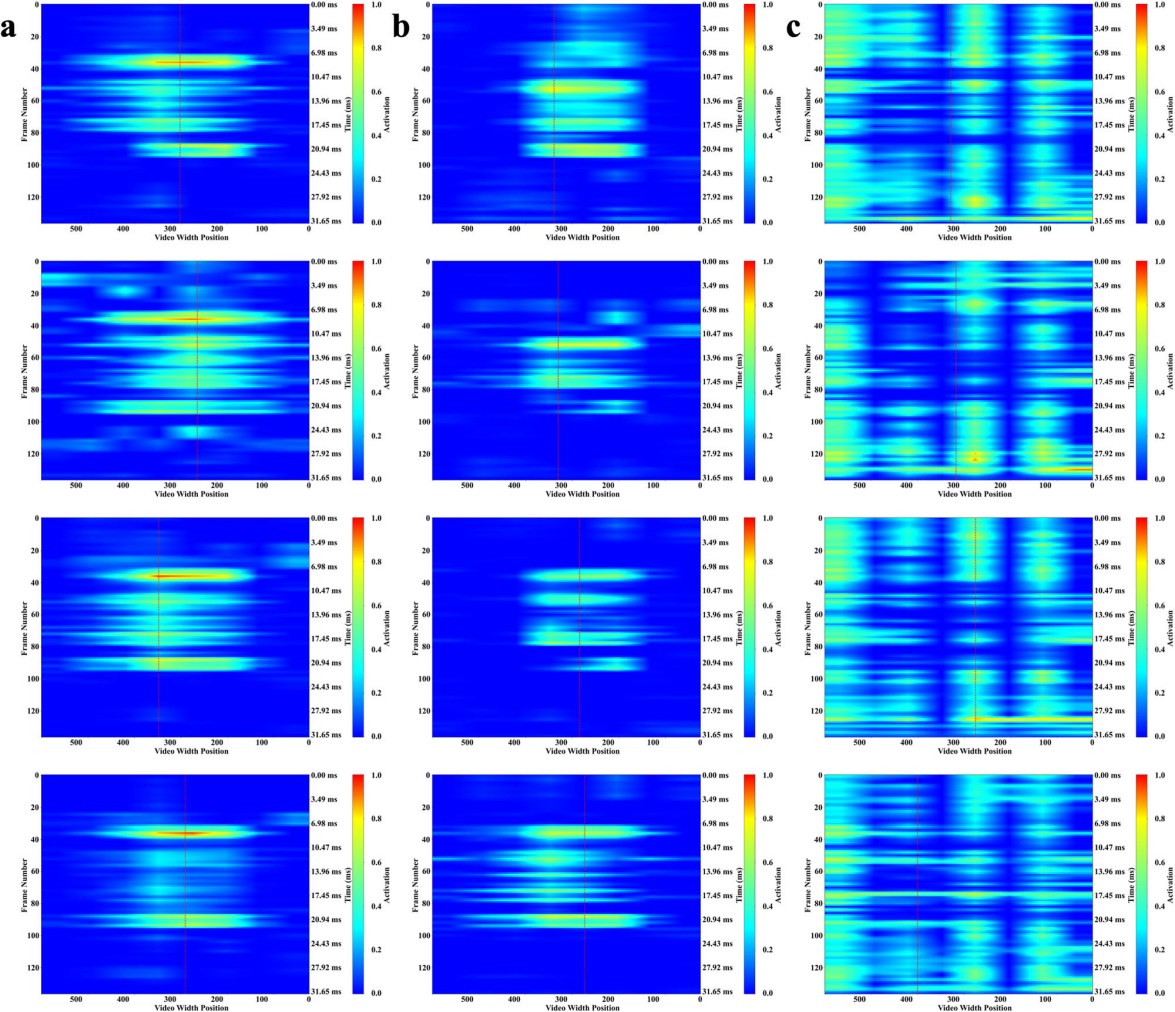
Selected examples of Grad-CAM revealing the area of the cornea and frame sequence that are most crucial for the model classification decision. **a** KC; **b** FFKC; **c** NC groups. The red dashed line represents the location of the cornea’s thinnest point. The red dashed line indicates the location of the thinnest point of the cornea.

In the spatial dimension (Fig. 4), where the clinically defined thinnest corneal point (red dashed line) was used as a reference anchor, the attention maps in the FFKC and KC cases exhibited sustained responses in the inferior or inferotemporal quadrants, where lesions were more likely to occur. Contrastingly, high-weight regions in the NC group were more centrally concentrated and more uniformly distributed. This spatial pattern is consistent with the pathological biomechanical features of KC, which are characterized by localized softening and structural nonuniformity.

In the temporal dimension, we integrated spatial attention into a time-importance curve and noted that the peaks were stably distributed from the first applanation to the maximum deformation and further to the second applanation. Notably, prominent peaks and a pronounced tail were additionally evident during the rebound phase. This indicates the presence of additional energy loss and viscous response during the deformation recovery process after unloading, an aspect that is easily overlooked when traditional static parameters are only considered at a single moment (such as the first flattening and maximum deformation). Our model became more sensitive to subtle and localized biomechanical abnormalities that are characteristic of KCS by incorporating rebound dynamics with comparable weights through spatiotemporal attribution. Especially in borderline cases, rebound-phase energy dissipation may serve as a valuable auxiliary indicator to guide closer follow-up or early intervention.

**Fig. 5:**
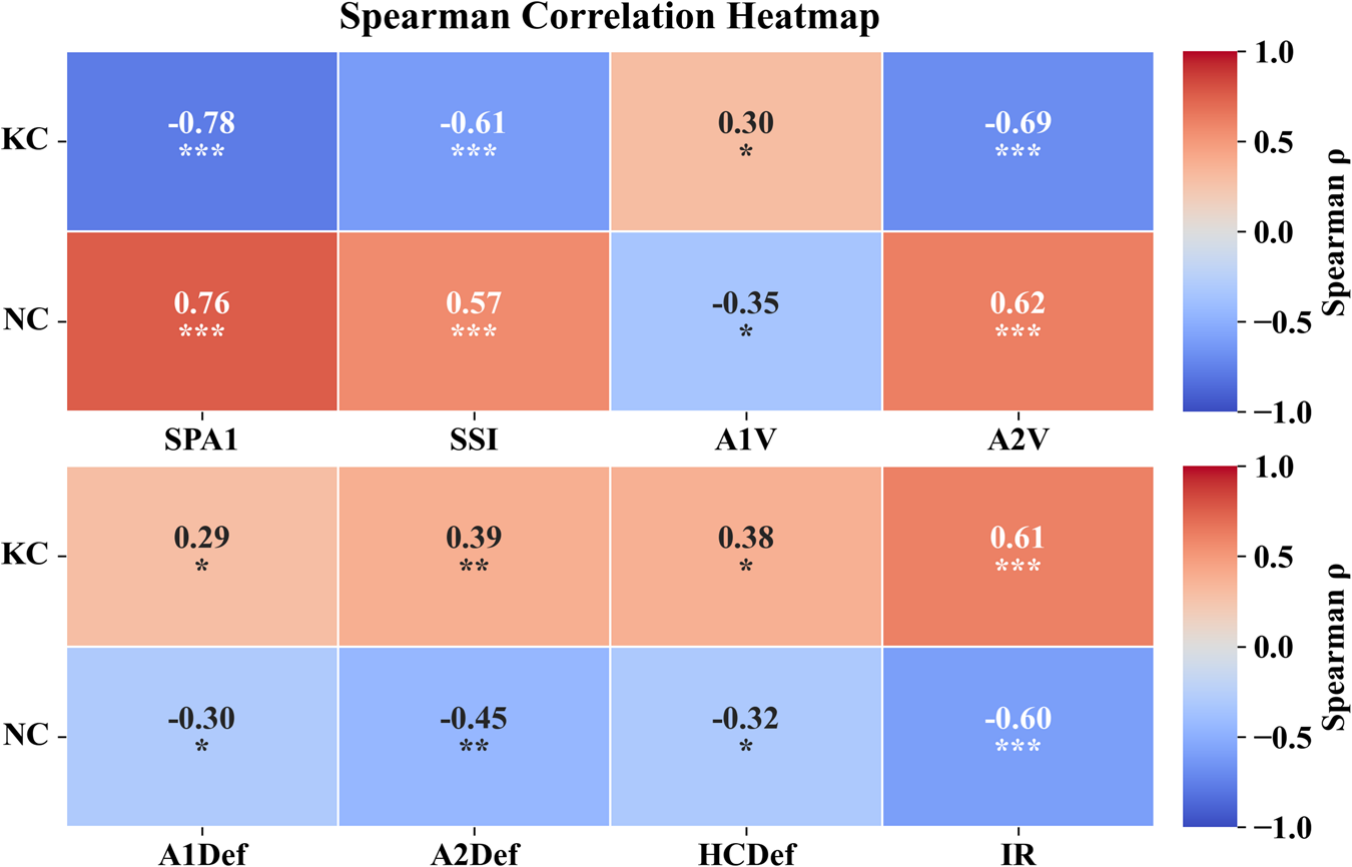
Spearman correlation heatmap between VST-derived features and biomechanical parameters (***p < 0.0001; **p < 0.001; *p < 0.01). A1V A1 Velocity, Corneal Apex Velocity at first Applanation, A2V A2 Velocity, Corneal Apex Velocity at second Applanation, A1Def A1 Deflection Amp, Deflection Amplitude at first Applanation, A2Def A2 Deflection Amp, Deflection Amplitude at second Applanation, HCDef HC Deflection Amp, Deflection Amplitude at Highest Concavity, IR Integrated Radius, Calculus of the radius of the reverse concavity, SPA1 Stiffness Parameter at first Applanation, SSI Stress-Strain Index.

In addition, we performed Spearman’s correlation analyses to evaluate the relationships between the features learned by the VST model and established biomechanical parameters (Fig. 5). SPA1 showed a strong negative correlation in the KC group (*r* = −0.78, *p* < 0.0001), whereas it exhibited a strong positive correlation in the NC group (*r* = 0.76, *p* < 0.0001). Similarly, SSI showed a strong negative correlation in the KC group (*r* = −0.61, *p* < 0.0001), but a moderate positive correlation in the NC group (*r* = 0.57, *p* < 0.0001). A2V also showed a strong negative correlation in the KC group (*r* = −0.69, *p* < 0.0001), and a strong positive correlation in the NC group (*r* = 0.62, *p* < 0.0001). In contrast, the Integrated Radius exhibited a strong positive correlation in the KC group (*r* = 0.61, *p* < 0.0001), but a strong negative correlation in the NC group (*r* = −0.60, *p* < 0.0001). Notably, A1V showed a moderate positive correlation in the KC group (*r* = 0.30, *p* < 0.01), whereas it showed a moderate negative correlation in the NC group (*r* = −0.35, *p* < 0.01). In addition, A1 Deflection Amp. showed a weak positive correlation in the KC group (*r* = 0.29, *p* < 0.01), whereas it showed a moderate negative correlation in the NC group (*r* = −0.30, *p* < 0.01). A2 Deflection Amp. demonstrated a moderate positive correlation in the KC group (*r* = 0.39, *p* < 0.001), but showed a moderate negative correlation in the NC group (*r* = −0.45, *p* < 0.001). HC Deflection Amp. also exhibited a moderate positive correlation in the KC group (*r* = 0.38, *p* < 0.01), whereas it showed a moderate negative correlation in the NC group (*r* = −0.32, *p* < 0.01). These results indicate that the features learned by the VST model may reflect key information related to biomechanics.

### AFM experimental validation of elasticity and viscoelasticity

**Fig. 6:**
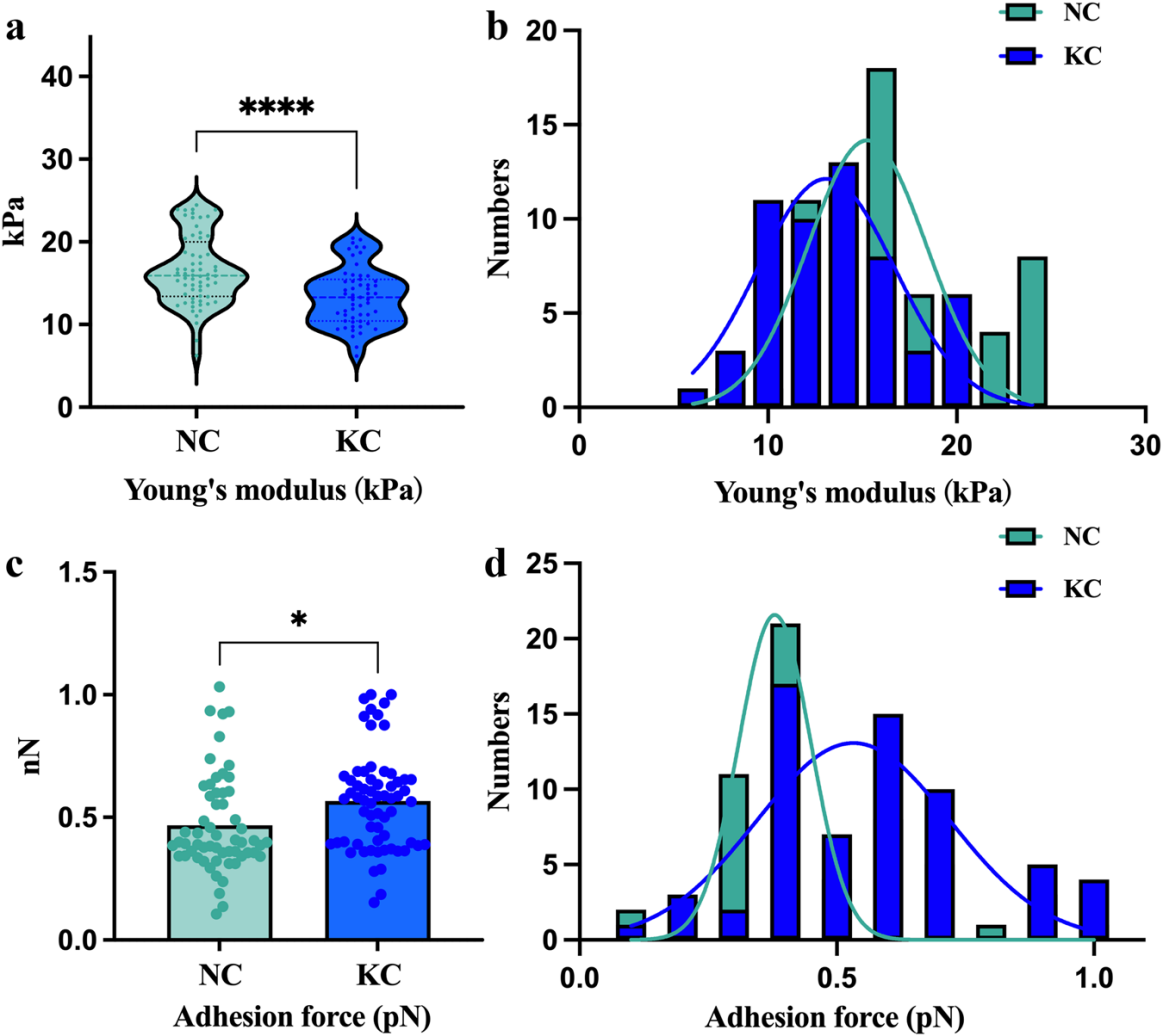
Analysis of the Young’s modulus and adhesion force in corneal stromal cells with NC and KC. **a** Violin plot illustrating the distribution of Young’s modulus. **b** Histogram comparison of the Young’s modulus among the two groups. **c** Scatter plots of the adhesion force with mean ± error bars across groups. **d** Histogram comparison of the adhesion force among the two groups.

We performed AFM nanoindentation and adhesion measurements to obtain supportive physical biomechanical evidence relevant to the altered rebound-phase viscoelastic behavior suggested by the VST model. The apparent Young’s modulus was lower in the KC group than in the NC group (13.09 ± 3.643 kPa vs. 15.26 ± 3.139 kPa, *P* < 0.0001, Fig. 6a, b), indicating decreased elasticity. Contrastingly, the adhesion force (Fig. 6c, d) was higher in the KC group (0.5321 ± 0.1875 pN) than in the NC group (0.3795 ± 0.07024 pN, *P* = 0.0258), indicating an improved adhesive effect after unloading. This softer and stickier microscopic phenotype supports the VST’s high-weight temporal attribution during the rebound phase: a lower Young’s modulus, along with a stronger adhesion tendency, may increase loading-unloading asymmetry and energy dissipation, which could manifest as amplified rebound-phase energy loss and a more pronounced trailing signal in video-based dynamic analysis.

Overall, three aspects supported the proposed model’s interpretability: spatial (attention concentrated in lesion-prone regions and around the thinnest point), temporal (contributions distributed across the full deformation process, with notable emphasis on the rebound phase), and physical experimental evidence (AFM-derived nanomechanical phenotypes). Collectively, these lines of evidence support the biological plausibility of the VST-derived spatiotemporal features in KCS detection and provide intuitive visual and experimental context to help clinicians understand the decision-making rationale of the model, thereby facilitating improved early screening.

### Ablation study

We conducted ablation experiments using various combinations of temporal dimensions and window sizes to elucidate their effects on model performance. Table 3 lists the resulting classification accuracies. The model achieved the highest accuracy of 96.49% when the temporal dimension was set to 136 frames, outperforming the 68-frame and 34-frame settings. This indicates that a longer temporal input captures more complete temporal dependencies and richer information, facilitating a more comprehensive analysis and ultimately enhancing classification performance.

**Table 3:** Ablation study on the temporal dimension of 3D tokens and temporal window size with Swin-B on Conus

temporal dimension	Window size	Precision
136	8 ×4 × 6	96.49%
68	4 × 4 × 6	93.86%
34	2 × 4 × 6	92.98%
136	8 × 4 × 6	96.49%
136	4 × 4 × 6	93.86%
136	2 × 4 × 6	92.98%
136	8 × 4 × 6	96.49%
68	8 × 4 × 6	92.11%
34	8 × 4 × 6	90.35%

The experiments were conducted with window sizes of 2 × 4 × 6, 4 × 4 × 6, and 8 × 4 × 6 when the temporal dimension was fixed at 136 frames. The model accuracy increased from 92.98% to 96.49% as the window size increased from 2 × 4 × 6 to 8 × 4 × 6. Thus, with sufficient temporal information, enlarging the window size helps the model better capture local spatiotemporal features. A larger window expands the attention coverage along the temporal axis, facilitating the model to integrate a longer-range temporal context within a single attention operation. This captures the discriminative dynamic representations of corneal deformation across extended time scales more effectively and ultimately improves performance.

Increasing the temporal length improved the accuracy from 90.35% to 96.49% with a fixed window size. However, the effect of window size exhibited the opposite trend under short-to-medium sequence settings: for 68 frames, window = 4 × 4 × 6 outperformed window = 8 × 4 × 6, and for 34 frames, window = 2 × 4 × 6 outperformed window = 8 × 4 × 6. Thus, an excessively large window can degrade performance when temporal information is limited. Increasing the window size yielded an overall performance that remains inferior to that achieved with long-sequence inputs when the temporal dimension is small, indicating that the completeness of temporal information is crucial for early KC diagnosis.

## Discussion

This study innovatively applied the VST to examine the entire process of corneal deformation under loading and subsequent unloading. Therefore, a new in vivo, force–deformation video-driven, and interpretable method was established for dynamic global spatiotemporal corneal biomechanical characterization. VST leverages 3D patch embedding and shifted-window self-attention to capture localized deformation regions in the spatial dimension and evolving deformation dynamics in the temporal dimension. Thus, this fundamentally overcomes the limitations of static, single-point, and global evaluations. This is unlike traditional analytical paradigms that extract static parameters from single-frame images at specific time points or locations. Applying VST to full-length dynamic deformation videos significantly enhanced the early KC identification, achieving an AUC of nearly 1.0, with a diagnostic accuracy (97.37%) significantly higher than that of BAD-D, CBI, and TBI (88.05%, 70.75%, and 88.75%, respectively; all *P* < 0.01).

Notably, interpretability analyses showed that the model consistently focused on the vicinity of the thinnest corneal point throughout the entire deformation process. The underscored suspected lesion region aligned with the clinically prevalent inferior or inferotemporal quadrant and matched the characteristic spatial pattern of focal, non-uniform biomechanical softening in KC^31,32^. Notably, an enhanced attention peak emerged during the rebound phase (around the second applanation), suggesting that post-unloading hysteresis and energy dissipation contribute prominently to differential diagnosis. This aspect is often overlooked in conventional studies that rely on discrete time-point sampling, such as the first applanation or maximum concavity^33^. In addition, AFM nanoindentation experiments revealed a reduced apparent Young’s modulus and elevated adhesion force in KC. This indicates a softer and more adhesive micromechanical phenotype, which provides supportive evidence consistent with the video-dynamics analysis. Collectively, VST can pinpoint the primary regions and time windows directly from video data, addressing the long-standing challenge for clinicians to extract pathogenic cues from dynamic deformation videos. It also provides novel quantitative evidence for early differential diagnosis and individualized intervention in KCS, with the potential to enhance diagnostic sensitivity and precision of personalized treatment selection in clinical decision-making.

VST performs end-to-end modeling of the dynamic mechanical information contained in the complete corneal deformation sequence, automatically learns global spatiotemporal patterns, and captures subtle biomechanical variations through multi-scale (local-to-global) characterization. This significantly enhances early identification (AUC of nearly 1.0). Contrastingly, traditional ML approaches (such as SVM and RF) rely on manual feature selection^18–20^. Although BAD-D, CBI, and TBI are composite indices^21,22^, they are essentially compressed characteristics derived from a limited number of time points or static features. Recent studies have also explored neural networks trained on curated biomechanical features or localized deformation descriptors^34^. From our perspective, the added diagnostic value of VST is primarily in its ability to directly analyze the entire dynamic deformation sequence, thereby capturing information in addition to static features and detecting subtle biomechanical alterations that may be missed when relying on manually selected features, which may be particularly important for early or suspicious cases. Collectively, these findings indicate that the dynamic deformation process itself, instead of static morphology alone, should receive greater emphasis in future early screening.

This study attempted to endow the model with supportive mechanistic explanations at three levels to transform AI from a black box predictor into an interpretable framework. First, at the feature level, we constructed a biomechanical progression trajectory based on the learned embedding characterization. This revealed a monotonic progression trend along the NC-FFKC-KC continuum from normal to subclinical and subsequently to manifest KC. This indicated that the potential feature learned by VST is consistent with disease progression and demonstrates interpretable continuity. Second, at the spatial level, we established a full-sequence contribution analysis scheme that precisely localizes the most informative time windows and spatial regions for early diagnosis. Grad-CAM spatial heatmaps revealed that the attention of the model was persistently concentrated around the thinnest point neighborhood in the FFKC and KC cases, which is highly consistent with the clinically prevalent inferotemporal lesion distribution. Third, at the temporal level, the time attribution curve identified key phases from the full-duration dynamics. This demonstrated that the post-unloading recovery hysteresis and trailing signals also carry considerable diagnostic value. Collectively, these findings indicate that the performance gain of the VST is not merely due to a stronger fitting capacity but rather arises from the synergistic effects of (i) a continuous mechanical progression trend, (ii) lesion-focused spatial attention, and (iii) heightened sensitivity to the rebound phase, all within a framework. Thus, this study deepens the understanding of the essence of corneal biomechanics and helps clinicians better leverage biomechanical information in decision-making. For example, a biomechanical progression trajectory derived from the AI-based learning could be implemented on relevant clinical devices to help clinicians rapidly and accurately determine disease status and stage, as shown in Fig. 2a.

Another crucial finding is that energy dissipation during the rebound phase plays a crucial role in the pathological biomechanics and sensitive diagnosis of the KCS. Previous studies have often averaged or overlooked this phase as it is subject to temporal accumulation and parameter compounding, focusing instead on discrete time points such as the first applanation or maximum concavity. However, true energy loss and asymmetry of recovery are more likely to manifest near the second applanation (that is, during rebound) as the cornea is a viscoelastic tissue. Herein, the sequential attribution analysis incorporated this phase with equal emphasis. Meanwhile, the AFM findings indicated a softer and more adhesive micromechanical phenotype in the KC samples. A reduced apparent Young’s modulus implies diminished elastic energy storage, whereas elevated adhesion suggests impeded energy release after unloading and greater dissipation. The current AFM findings should be regarded as supportive physical experimental evidence consistent with the rebound-phase abnormalities suggested by the VST model. Overall, in addition to categorical classification, this study also provides multi-level quantitative information, including probability calibration, a continuous biomechanical progression trajectory derived from the embedding space, and dynamic indicators obtained from spatiotemporal attribution analysis, such as rebound-phase energy dissipation. Once validated, these features may serve as interpretable quantitative biomarkers for identifying biomechanical instability in borderline cases when used in conjunction with corneal tomography and slit-lamp examination.

Recent physics-based corneal biomechanical modeling studies have provided an important interpretative context for understanding these video-derived features. As a viscoelastic tissue, the cornea dissipates part of the input mechanical energy during the loading and unloading processes, resulting in viscoelastic damping and hysteresis^35^. Normal corneas generally exhibit more pronounced hyperelastic behavior and therefore tend to recover more rapidly after external loading, with weaker hysteretic effects. By contrast, when the material properties of the cornea become abnormal, particularly when the organization and distribution of collagen fibers are disrupted, its hyperelastic behavior may decline, whereas viscoelasticity and time-dependent behavior may increase, ultimately manifesting as slower rebound, greater hysteresis, and abnormal energy dissipation^36–38^. Finite-element analyses and shear-wave elastography studies have shown that the dynamic corneal responses are jointly determined by intrinsic material properties, viscoelastic behavior, geometric factors, and IOP, whereas some studies suggest that tissue viscoelasticity may play a particularly dominant contribution^39–42^. In addition, finite-element modeling studies have reported that KC is associated with reduced shear-wave velocity and increased apical corneal deformation, potentially linking focal thinning and softening to abnormal spatiotemporal biomechanical behavior^43^. Notably, Lombardo et al. extracted deformation profiles and fitted viscoelastic models to estimate corneal stiffness (kc, N/m), achieving strong diagnostic performance for KC detection and providing important mechanistic context^26,27^. On this basis, we performed Spearman correlation analysis and determined that the features learned by the VST model were significantly associated with corneal biomechanical information. Overall, these findings support the idea that the spatiotemporal features learned by VST, especially those related to the rebound phase, may reflect physical biomechanical alterations in early ectatic disease, including corneal softening, impaired viscoelastic damping, altered hysteretic behavior, and abnormal energy dissipation. However, compared with explicit biomechanical parameters derived from physics-based modeling, these AI-learned features remain relatively limited in terms of direct interpretability with respect to specific mechanical parameters. Therefore, in future work, we plan to integrate explicit biomechanical parameters with AI-derived features to establish an analytical framework that better balances physical interpretability and predictive performance.

Although the VST model achieved near-perfect AUC values and maintained strong diagnostic performance in the external validation, calibration errors increased for KC and FFKC. This indicates that, in practice, potential sources of variability may include differences in device calibration status, air-puff conditions, image acquisition quality, patient alignment and fixation stability, as well as population composition across centers. In the current study, we attempted to reduce acquisition-related variability by applying an identical preprocessing and standardization pipeline across all cohorts. However, borderline cases, with a biomechanical phenotype that lies between NC and KC, may be more sensitive to confidence instability near the decision boundary. Therefore, the clinical translation of video-based biomechanical AI models requires not only strong diagnostic performance but also domain adaptation to minimize statistical differences in feature distributions across centers, as well as through stable cross-center probability calibration to decrease the impact of regional variability on model output. In addition, future translation should also consider practical and regulatory considerations, including standardized data acquisition, model version control, quality-control procedures, and integration with existing clinical diagnosis and treatment workflows, to support safe, stable, and consistent clinical implementation.

This study has some limitations. First, Corvis ST relies on a single-axis imaging setup and a fixed air-puff paradigm, which provides limited access to non-axial anisotropy and a three-dimensional lamellar network. Therefore, integrating complementary modalities such as OCT is required to capture richer structural information and strengthen structural and mechanical characterization. Second, Grad-CAM is a post-hoc visualization tool that can improve interpretability but does not constitute causal evidence. In future studies, we plan to attempt to establish associations between physical mechanical characterizations and AI-derived features within the same samples, in combination with experimental validation and finite-element modeling, to provide stronger support for cross-modal biomechanical interpretation. Third, the discriminative information learned by the model in the current study may reflect the combined effects of disease-related biomechanical abnormalities and their accompanying structural alterations (such as CCT). In future studies, we will adopt strategies such as covariate adjustment to more fully elucidate the independent contribution of intrinsic material biomechanical properties. Notably, the model is better positioned as a clinical decision-support tool rather than a replacement for clinician judgment. The current external validation is encouraging but remains limited, and prospective validation in larger cohorts and multicentre real-world clinical settings is required to comprehensively assess the robustness and translational value of the proposed technique.

In conclusion, this study introduced a novel VST-based framework that enables in vivo, force–deformation video-driven, and interpretable characterization of corneal dynamic biomechanics. This framework demonstrated significant advantages in the early differential diagnosis of keratoconus suspects. The model’s persistent spatial focus on the thinnest point neighborhood and its high temporal attribution to the rebound phase are highly compatible with the AFM-derived micromechanical phenotype of KC, which is softer and more adhesive. This highlights the crucial role of rebound-phase energy dissipation in the pathological biomechanics of KC from a dynamic perspective. This framework offers novel concepts, methods, and quantifiable evidence to support early differential diagnosis, longitudinal monitoring, and individualized intervention for KC. It also provides a generalizable methodological path for the intelligent analysis of video-level biomechanical signals. Future advancements in the integration of 3D imaging modalities and enhanced calibration strategies have the potential to further enhance the diagnostic sensitivity and precision of personalized treatment selection in real-world clinical decision-making.

## Methods

### Participants, consent, and data collection

Following a multicentre study design, participants from two hospitals were enrolled for algorithm development testing and an external validation cohort. Data from Nankai University Affiliated Tianjin Eye Hospital was used for model development and testing, whereas data from Shaanxi Eye Hospital was used as an independent external validation cohort. The Ethics Committee of Nankai University Affiliated Tianjin Eye Hospital (No. 2022021) and the Ethics Committee of Shaanxi Eye Hospital (No. KJLL-Z-H-2025001) all approved the study protocol, and the study was conducted in accordance with the principles of the Declaration of Helsinki. All participants provided informed consent.

Two experienced corneal specialists (Y.W. and J.L.) independently classified participants. Participants whose eyes met the KC diagnostic criteria were assigned to the KC group. The inclusion criteria for KC included a history of myopia or astigmatism, corrected distant visual acuity (CDVA) < 20/20; at least one positive slit-lamp finding (corneal stromal thinning, anterior protrusion, Vogt’s striae, Fleischer’s ring, or Munson’s sign); and abnormal corneal tomography, such as a superior–inferior asymmetry >3.0 D within a 3 mm radius of the anterior corneal surface or an interocular difference in central anterior surface curvature >1.0 D. Participants with clinical KC signs in only one eye while the contralateral eye showed normal findings on slit-lamp examination, retinoscopy, and corneal tomography (superior–inferior asymmetry <1.45 D within a 3 mm radius; absence of asymmetric bow-tie, focal, or inferior steepening patterns; CDVA ≥ 20/20), were classified as the forme fruste KC (FFKC) group. The third group consisted of normal control (NC), defined by CDVA ≥ 20/20 and no ocular abnormalities other than refractive errors and normal corneal tomography. Before examination, participants were required to discontinue soft contact lens wear for ≥2 weeks and rigid contact lens wear for ≥4 weeks. Patients who had a history of ocular surgery or trauma, ocular diseases other than refractive error and primary KC, connective tissue disorders, or systemic diseases were excluded from the study.

Corneal topography was performed using a three-dimensional anterior segment analysis and diagnosis system (Pentacam HR, Oculus, Wetzlar, Germany). Corneal dynamic deformation videos were acquired using the Corneal Visualization Scheimpflug Technology (Corvis ST; Oculus, Wetzlar, Germany). The system induces corneal deformation with an air puff and conducts high-speed Scheimpflug imaging at 4330 frames per s within an 8 mm horizontal meridian. Each video sequence had a 576 × 192 pixel spatial resolution and a 31.88 ms duration. Three repeated measurements were obtained for each eye, and the analysis included only recordings with an examination quality status of “QS = OK”. In addition to the deformation videos, clinical data were collected, including age, sex, uncorrected visual acuity, corrected distance visual acuity (CDVA), objective and subjective refraction, noncontact intraocular pressure, corneal tomography, and findings from slit lamp and fundus examinations.

### Video preprocessing

All videos underwent unified artifact suppression and background normalization to improve the robustness and repeatability of model learning. Let ${v=\left\{{v}_{i}\right\}}_{i=1}^{N}$ denote the set of input videos, where ${v}_{i}$ represents the i-th sample. Each video ${v}_{i}$ was first decoded into a sequence of frames ${\left\{{I}_{t}\right\}}_{t=1}^{{T}_{i}}$, where ${T}_{i}$ is the number of frames, and each frame ${I}_{t}\in {{\mathbb{R}}}^{H\times W\times 3}$ denotes an RGB image with height H and width W. The temporal length was standardized to 136 frames by removing invalid leading and trailing frames, ensuring temporal alignment across samples.

To suppress stable artifacts such as watermarks and localized stray light, a filling strategy was applied within predefined regions of interest (ROIs), denoted as ${\left\{{{\mathcal{R}}}_{k}\right\}}_{k=1}^{K}$, where each ${{\mathcal{R}}}_{k}\subseteq \left\{1,...,H\right\}\times \left\{1,...,W\right\}$ represents a spatial pixel region. For each frame ${I}_{t}$, the image was evenly divided into three segments along the horizontal axis. Let ${\left\{{{\mathscr{S}}}_{j}\right\}}_{j=1}^{3}$ denote these segments, and ${\mu }_{t,j}$ the mean grayscale intensity of segment ${{\mathcal{S}}}_{j}$, computed via a grayscale conversion function ${\mathscr{G}}\left(\cdot \right)$. The background intensity ${c}_{t}\in {{\mathbb{R}}}^{3}$ was then estimated as the average RGB value over the two segments with lower mean intensities:

1 $${c}_{t}=\frac{1}{\left|{\Omega }_{t}^{{dark}}\right|}\mathop{\sum }\limits_{p\in {\Omega }_{t}^{dark}}{I}_{t}\left(p\right)$$

where ${\varOmega }_{t}^{{dark}}={{\mathcal{S}}}_{{j}^{1}}\cup {{\mathcal{S}}}_{{j}^{2}}$ corresponds to the union of the two segments with smaller ${\mu }_{t,j}$, and p denotes a pixel location.

For each ROI ${{\mathcal{R}}}_{k}$, a binary mask ${M}_{t}\left(p\right)\in \left\{\mathrm{0,1}\right\}$ was constructed based on grayscale intensity:

2 $$Mt(p)=I{\mathscr{(}}{\mathcal{G}}{\mathscr{(}}It(p)) > 80)$$

where $I\left(\cdot \right)$ is the indicator function. Pixels satisfying the mask condition (i.e., ${M}_{t}\left(p\right)=1$) were replaced by the estimated background value ${c}_{t}$, while other pixels remained unchanged. **Algorithm 1** showed the details of the preprocessing steps. In addition, each frame was normalized using the channel-wise mean and standard deviation, according to the following formula:

3 $${I}^{{\prime} }=\frac{I-\mu }{\sigma }$$

where, $\mu =\left[\mathrm{123.675,116.28,103.53}\right],$ σ $=\left[\mathrm{58.395,57.12,57.375}\right]$. Normalization was applied separately to each RGB channel, while the input images were retained in RGB format. Subsequently, training and validation were performed using the original unaltered video data to preserve, as much as possible, the authenticity and integrity of the dynamic corneal response.

#### Algorithm 1

Data Preprocessing

**Input:** Video set ${\mathscr{V}}={\mathscr{\{}}{v}_{i}{\}}_{i=1}^{N}$

**Output:** Processed videos $\{{\widetilde{v}}_{i}{\}}_{i=1}^{N}$

**for** i←1 **to** N **do**

│ $$\{{I}_{t}{\}}_{t=1}^{{T}_{i}}\leftarrow {ExtractFrames}\left({v}_{i}\right);$$

│ $${f}_{i}\leftarrow {FPS}\left({v}_{i}\right);$$

│ **for** t←1 **to** ${T}_{i}$ **do**

│ │ $${\varOmega }_{i,t}^{{dark}}\leftarrow {{Top}}_{2/3}\left({I}_{t}\right);$$          // darkest pixels

│ │ $${c}_{i,t}\leftarrow 1/\left|{\varOmega }_{i,t}^{{dark}}\right|\mathop{\sum }\limits_{p\in {\varOmega }_{i,t}^{{dark}}}{I}_{t}\left(p\right);$$

│ │ **for** k←1 **to** K **do**

│ │ │ **for each** $p\in {{\mathscr{R}}}_{k}$ **do**

│ │ │ │ $${G}_{i,t}\left(p\right){\mathscr{\leftarrow }}{\mathscr{G}}\left({I}_{t}\left(p\right)\right);$$

│ │ │ │ $${M}_{i,t}\left(p\right){\mathbb{\leftarrow }}{\mathbb{I}}\left({G}_{i,t}\left(p\right) > 80\right);$$

│ │ │ │ **if** $${M}_{i,t}\left(p\right)=1$$ **then**

│ │ │ │ └ $${I}_{t}\left(p\right)\leftarrow {c}_{i,t};$$

│ $${\widetilde{v}}_{i}\leftarrow {Encode}\left(\{{I}_{t}{\}}_{t=1}^{{T}_{i}},{f}_{i}\right);$$

│ $${\widetilde{v}}_{i}\leftarrow {Crop}\left({\widetilde{v}}_{i},576\times 192,\left(\mathrm{0,8}\right)\right);$$

└ $${Save}\left({\widetilde{v}}_{i}\right);$$

### Video swin transformer (VST) model

**Fig. 7:**
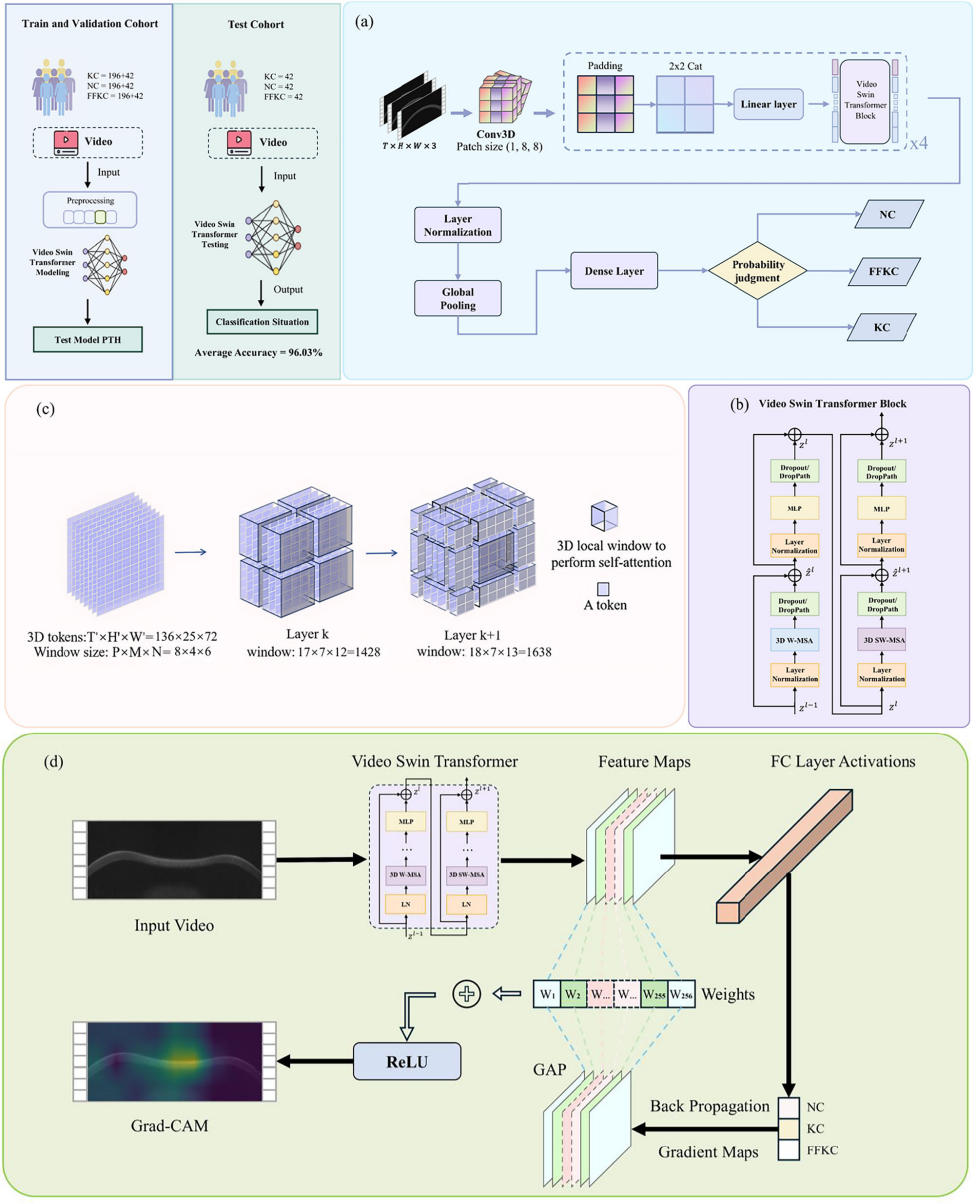
Overview of the Video Swin Transformer model and the interpretability analysis pipeline. **a** Overall framework of the Video Swin Transformer model; **b** Basic block structure with alternating W-MSA/SW-MSA and MLP modules; **c** 3D sliding-window self-attention mechanism; **d** Schematic illustration of the Grad-CAM–based heatmap generation process.

VST is a recently proposed model that extends the transformer framework to video classification. Herein, we present the first adaptation of an enhanced transformer framework for the global spatiotemporal dynamic biomechanical characterization of corneal deformation videos. Each corneal deformation video consisted of 136 frames with a spatial resolution of 576 × 192 (width × height). Each frame was partitioned into non-overlapping patches across the temporal and spatial dimensions (T = 136, H = 192, W = 576), with patch sizes of (1, 8, and 8), corresponding to the temporal, height, and width dimensions, respectively. This resulted in T/1 × H/8 × W/8 × 192 tokens. Each token was a 192-dimensional vector (1 × 8 × 8 × 3 = 192) representing the RGB pixel values within a patch. The Swin Transformer module serves as a spatiotemporal feature extractor by extending attention computation from the spatial domain to the joint spatiotemporal domain by leveraging the inherent spatiotemporal characteristics of video data. Spatially, the model captured inter-frame and intra-frame spatial dependencies while temporally modeling dynamic deformation patterns across frames. Local inductive biases were introduced via spatiotemporal factorization and non-overlapping windows through the 3D shifted-window multihead self-attention (3DSW-MSA) mechanism. This design enabled the model to learn locality, hierarchical representations, and translation invariance, thereby progressively extracting corneal biomechanical features from local to global scales. Finally, the extracted features were mapped to the classification outputs using a dense layer. Additionally, contribution scores from various temporal segments and spatial regions were integrated to quantify their relative importance in the final classification. Figure 7a illustrates the architecture of the proposed model.

### VST block

The VST Block primarily includes a 3D window-based multihead self-attention (W-MSA) module, a 3D shifted-window multihead self-attention (SW-MSA) module, and a multilayer perceptron (MLP) module (Fig. 7 b). Two alternating structures were used for each block. First, 3DW-MSA (window MSA) was applied to calculate the local representations within each spatiotemporal window. This is followed by a 3DSW-MS (shifted-window MSA), which provides information exchange across neighboring windows and facilitates global context modeling. These two structures are used in pairs to progressively capture local and global spatiotemporal dependencies. The corresponding calculations were as follows:

4 $$\begin{array}{c}{\hat{z}}^{l}=3{DW\hbox{-}MSA}({LN}({z}^{l-1}))+{z}^{l-1}\\ {z}^{l}={FFN}({LN}({\hat{z}}^{l}))+{\hat{z}}^{l},\\ {\hat{z}}^{l+1}=3{DSW\hbox{-}MSA}({LN}({z}^{l}))+{z}^{l},\\ {z}^{l+1}={FFN}({LN}({\hat{z}}^{l+1}))+{\hat{z}}^{l+1},\end{array}$$

where ${\hat{z}}^{l}$ denotes the output of the 3D (S)W-MSA module in block 1 and ${z}^{l}$ denotes the output of the MLP module in block 1.

### 3D sliding-window self-attention mechanism

The VST block uses a 3D sliding-window self-attention mechanism in which local attention is computed along the spatial dimensions, whereas global temporal dependencies are modeled along the time dimension (Fig. 7c). In particular, the input video is first partitioned into a set of tokens arranged in a $T\times H\times W$ grid, where T denotes the temporal dimension (number of frames), and H and W denote the spatial height and width, respectively. The window size was defined as $P\times M\times N$(P=8, M=4, N=6). Self-attention is calculated within each window, facilitating localized attention and the effective extraction of spatially local features from the corneal deformation video sequence. Subsequently, cross-window connections are established via window overlapping or shifting strategies, which facilitate information exchange across neighboring windows and enable the modeling of correlations across various spatiotemporal regions. Using this hierarchical attention mechanism, the model captures corneal biomechanical features ranging from local to global scales across the entire spatiotemporal domain of the video.

### 3D relative position encoding

In the self-attention computation process, 3D relative position encoding (3D RPE) is incorporated to enable the learning of global relationships by capturing spatiotemporal dependencies across various video frames. This mechanism improves the ability of the model to analyze and represent complex video data. The formulation is provided as follows:

5 $${Attention}(Q,K,V)={softmax}\left(\frac{Q{K}^{T}}{\sqrt{{d}_{k}}}\right)V$$

$$Q=X{W}^{Q}$$

$$K=X{W}^{K}$$

$$V=X{W}^{V}$$

where Q is the query, K is the key, and V is the key value. Q, K, and V are obtained by applying linear transformations to the input matrix X. ${W}^{Q}$, ${W}^{K}$, and ${W}^{V}$ are the parameter matrices. Each is multiplied by the input matrix X, which is equivalent to performing a linear transformation.

### Grad Class Activation Map

Gradient-weighted Class Activation Mapping (Grad-CAM) is a class-discriminative localization approach used to visualize the decision-making process of deep learning models. This study used Grad-CAM to visualize the average attention in diagnostic regions and assess their contributions to the diagnosis to quantify the dynamic biomechanical characteristics of the cornea throughout the force-induced deformation process (Fig. 7d). The corneal video was input into the VST model, and the output of the last normalized layer was selected as the target layer. The features in this layer are weighted and combined with the activation values to generate a heatmap using the gradient information propagated backward, highlighting the key regions in which the model focuses on the decision-making process. Specifically, the contribution of various regions to the final classification was calculated in the spatial dimension to identify the class-discriminative areas of the cornea (e.g., locations that are crucial). The contributions of the features in the temporal dimension at varying time points to the final classification were calculated to identify the most critical moments (e.g., the moment of maximum deformation). The importance of each region and time point to the final classification is visually represented by generating attention maps, where red regions indicate a high contribution to the classification and blue regions indicate a lower contribution.

### Atomic force microscopy detection of Young’s modulus and adhesion force

Sample sources: Corneal tissue samples were collected from patients with KC undergoing deep lamellar keratoplasty (DLK) and from individuals with normal corneas undergoing femtosecond laser small incision lenticule extraction (SMILE). The Ethics Committee of Nankai University affiliated with Tianjin Eye Hospital, approved all tissue collection procedures (No. KY2025021), and all patients provided informed consent.

Primary cell isolation and culture: Fresh tissues were transferred into a 15-mL centrifuge tube and incubated with 2 mL of type I collagenase working solution (2 mg/ml, Sigma) in a 37 °C incubator with shaking for 2–3 h. Digestion was terminated by adding complete culture medium (DMEM/F12 medium, Gibco, supplemented with 10% fetal bovine serum, Gibco, and 1% penicillin-streptomycin mixture, Invitrogen). The cells were centrifuged at 1000 rpm for 3 min, resuspended in fresh complete medium, and cultured in a 37 °C, 5% CO₂ incubator. Cells from passages 3–5 were used in the experiments.

Atomic force microscopy (AFM) nanoindentation and adhesion measurements: A Bioscope Resolve AFM system (Bruker, Santa Barbara, CA, USA) was installed on an Eclipse Ti2-U inverted fluorescence microscope (Nikon, Tokyo, Japan). The system was equipped with a heated sample stage to maintain the cell-culture temperature at 37 °C. A PeakForce QNM-Live Cell probe was utilized to measure the deflection sensitivity in air via the interaction between the probe and the hard substrate. The bending stiffness of the probe was measured based on its thermal equilibrium state when it was away from the sample. Thermal tuning was conducted in the liquid to measure the deflection sensitivity based on the calculated stiffness values. Nanomechanical and morphological mapping involved scanning a 50 × 50 μm area with a resolution of 128 × 128 points. The vertical ramp size was 5 μm, the vertical piezo velocity was 203 μm/s, the trigger threshold was 20 nm, the trigger force was <0.5 nN, and the scan rate was 20.3 Hz. Force–displacement curves were fitted using the Hertz model to obtain the apparent (effective) Young’s modulus of the cell surface, from which the cellular Young’s modulus was further calculated. At least 600 distinct locations were examined per sample, and the mean value was reported as the final result. Data were analyzed using Nanoscope Analysis software (version 3.0; Bruker, Santa Barbara, CA, USA).

### Statistical analysis

Statistical analyses were conducted using SPSS (version 26.0; IBM, Armonk, NY, USA). Quantitative data are presented as the mean and standard error of the mean. Normality was evaluated using the Kolmogorov–Smirnov test. Between-group comparisons were conducted using a one-way analysis of variance (ANOVA). All tests were two-sided, and a *P*-value < 0.05 was considered statistically significant. Receiver operating characteristic (ROC) curve analysis was used to assess the diagnostic performance of the BAD-D, CBI, and TBI for FFKC. Additionally, the optimal cutoff values were determined using the Youden index. Confusion matrices were utilized to calculate the accuracy, sensitivity, specificity, precision, recall, and F1 score to quantify the overall performance of each method during the classification task.

## Data Availability

The datasets generated and/or analyzed during the current study are not publicly available due to compliance with patient privacy protection, but are available from the corresponding author on reasonable request (wangyan7143@vip.sina.com).

## References

[1] Thomasy, S. M., Leonard, B. C., Greiner, M. A., Skeie, J. M. & Raghunathan, V. K. Squishy matters - corneal mechanobiology in health and disease. *Prog. Retin. Eye Res.* **99**, 101234 (2024). 38176611 10.1016/j.preteyeres.2023.101234 PMC11193890

[2] Singh, RB. et al. Keratoconus. *Nat. Rev. Dis. Primers* **10**, 81 (2024). 39448666 10.1038/s41572-024-00565-3

[3] Vinciguerra, R., Ambrósio, R. Jr, Roberts, C. J., Azzolini, C. & Vinciguerra, P. Biomechanical characterization of subclinical keratoconus without topographic or tomographic abnormalities. *J. Refract. Surg.* **33**, 399–407 (2017). 28586501 10.3928/1081597X-20170213-01

[4] Chen, X. et al. Screening of sensitive in vivo characteristics for early keratoconus diagnosis: a multicenter study. *Front. Bioeng. Biotechnol.* **11**, 1158299 (2023). 37600309 10.3389/fbioe.2023.1158299 PMC10436515

[5] Chong, J. & Dupps, W. J. Jr Corneal biomechanics: measurement and structural correlations. *Exp. Eye Res.* **205**, 108508 (2021). 33609511 10.1016/j.exer.2021.108508 PMC8046161

[6] Andreassen, T. T., Simonsen, A. H. & Oxlund, H. Biomechanical properties of keratoconus and normal corneas. *Exp. Eye Res.* **31**, 435–441 (1980). 7449878 10.1016/s0014-4835(80)80027-3

[7] Lombardo, M. et al. Biomechanics of the anterior human corneal tissue investigated with atomic force microscopy. *Invest. Ophthalmol. Vis. Sci.* **29**, 1050–7 (2012). 10.1167/iovs.11-8720 22266511

[8] Labate, C. et al. Multiscale investigation of the depth-dependent mechanical anisotropy of the human corneal stroma. *Invest. Ophthalmol. Vis. Sci.* **56**, 4053–60 (2015). 26098472 10.1167/iovs.15-16875 PMC4477262

[9] Labate, C., De Santo, M. P., Lombardo, G. & Lombardo, M. Understanding of the viscoelastic response of the human corneal stroma induced by riboflavin/UV-a cross-linking at the nano level. *PLoS ONE.* **1**, e0122868 (2015). 10.1371/journal.pone.0122868 PMC4382164 25830534

[10] Labate, C., Lombardo, M., Lombardo, G. & De Santo, M. P. Biomechanical strengthening of the human cornea induced by nanoplatform-based transepithelial riboflavin/UV-A corneal cross-linking. *Invest. Ophthalmol. Vis. Sci.* **1**, 179–184 (2017). 10.1167/iovs.16-20813 28114577

[11] Scarcelli, G., Besner, S., Pineda, R. & Yun, S. H. Biomechanical characterization of keratoconus corneas ex vivo with Brillouin microscopy. *Invest. Ophthalmol. Vis. Sci.* **55**, 4490–4495 (2014). 24938517 10.1167/iovs.14-14450 PMC4109405

[12] Raoux, C., Chessel, A., Mahou, P., Latour, G. & Schanne-Klein, M. C. Unveiling the lamellar structure of the human cornea over its full thickness using polarization-resolved SHG microscopy. *Light Sci. Appl.* **12**, 190 (2023). 37528091 10.1038/s41377-023-01224-0 PMC10394036

[13] Morishige, N. et al. Second-harmonic imaging microscopy of normal human and keratoconus cornea. *Invest. Ophthalmol. Vis. Sci.* **48**, 1087–1094 (2007). 17325150 10.1167/iovs.06-1177 PMC1894888

[14] Hallahan, K. M., Sinha Roy, A., Ambrosio, R. Jr, Salomao, M. & Dupps, W. J. Jr Discriminant value of custom ocular response analyzer waveform derivatives in keratoconus. *Ophthalmology* **121**, 459–468 (2014). 24289916 10.1016/j.ophtha.2013.09.013 PMC4031747

[15] Wang, W., Du, S. & Zhang, X. Corneal deformation response in patients with primary open-angle glaucoma and in healthy subjects analyzed by Corvis ST. *Invest. Ophthalmol. Vis. Sci.* **56**, 5557–5565 (2015). 26305527 10.1167/iovs.15-16926

[16] Randleman, JB. et al. Subclinical keratoconus detection and characterization using motion-tracking Brillouin microscopy. *Ophthalmology* **131**, 310–321 (2024). 37839561 10.1016/j.ophtha.2023.10.011 PMC11117393

[17] Feng, X., Li, G. Y., Jiang, Y., Shortt-Nguyen, O. & Yun, S. H. Optical coherence elastography measures mechanical tension in the lens and capsule. *Acta Biomater.* **199**, 252–261 (2025). 40319993 10.1016/j.actbio.2025.05.009 PMC13196500

[18] Saad, A. & Gatinel, D. Topographic and tomographic properties of forme fruste keratoconus corneas. *Invest. Ophthalmol. Vis. Sci.* **51**, 5546–5555 (2010). 20554609 10.1167/iovs.10-5369

[19] Arbelaez, M. C., Versaci, F., Vestri, G., Barboni, P. & Savini, G. Use of a support vector machine for keratoconus and subclinical keratoconus detection by topographic and tomographic data. *Ophthalmology* **119**, 2231–2238 (2012). 22892148 10.1016/j.ophtha.2012.06.005

[20] Herber, R., Pillunat, L. E. & Raiskup, F. Development of a classification system based on corneal biomechanical properties using artificial intelligence predicting keratoconus severity. *Eye Vis.* **8**, 21 (2021). 10.1186/s40662-021-00244-4 PMC8167942 34059127

[21] Ambrósio, R. Jr. Optimized artificial intelligence for enhanced ectasia detection using Scheimpflug-based corneal tomography and biomechanical data. *Am. J. Ophthalmol.* **251**, 126–142 (2023). 36549584 10.1016/j.ajo.2022.12.016

[22] Vinciguerra, R. et al. Detection of keratoconus with a new biomechanical index. *J. Refract. Surg.* **32**, 803–810 (2016). 27930790 10.3928/1081597X-20160629-01

[23] Tan, Z. et al. Artificial intelligence-based diagnostic model for detecting keratoconus using videos of corneal force deformation. *Transl. Vis. Sci. Technol.* **11**, 32 (2022). 36178782 10.1167/tvst.11.9.32 PMC9527334

[24] Chen, X. et al. Localized corneal biomechanical alteration detected in early keratoconus based on corneal deformation using artificial intelligence. *Asia Pac. J. Ophthalmol.* **12**, 574–581 (2023). 10.1097/APO.0000000000000644 37973045

[25] Tan, Z. et al. Unsupervised corneal contour extraction algorithm with shared model for dynamic deformation videos: improving accuracy and noise resistance. *Biomed. Eng. Online* **23**, 4 (2024). 38191452 10.1186/s12938-023-01188-7 PMC10775613

[26] Lombardo, G. et al. Accuracy of an air-puff dynamic tonometry biomarker to discriminate the corneal biomechanical response in patients with keratoconus. *Cornea* **1**, 315–322 (2024). 10.1097/ICO.0000000000003377 37964435

[27] Lombardo, G., Bernava, G. M., Serrao, S. & Lombardo, M. Theranostic-guided corneal cross-linking: preclinical evidence on a new treatment paradigm for keratoconus. *J Biophotonics* **15**, e202200218 (2022). 36059083 10.1002/jbio.202200218

[28] Arizcuren, A. R. & Consejo, A. A method for visualizing corneal dynamics through pixel intensity tracking. *Transl. Vis. Sci. Technol.* **14**, 24 (2025). 40504568 10.1167/tvst.14.6.24 PMC12169463

[29] Shamshad, F. et al. Transformers in medical imaging: a survey. *Med. Image Anal.* **88**, 102802 (2023). 37315483 10.1016/j.media.2023.102802

[30] Li, J. et al. Transforming medical imaging with Transformers? A comparative review of key properties, current progresses, and future perspectives. *Med. Image Anal.* **85**, 102762 (2023). 36738650 10.1016/j.media.2023.102762 PMC10010286

[31] Santodomingo-Rubido, J. et al. Keratoconus: an updated review. *Cont. Lens Anterior Eye* **45**, 101559 (2022). 34991971 10.1016/j.clae.2021.101559

[32] Scarcelli, G., Besner, S., Pineda, R., Kalout, P. & Yun, S. H. In vivo biomechanical mapping of normal and keratoconus corneas. *JAMA Ophthalmol.* **133**, 480–482 (2015). 25611213 10.1001/jamaophthalmol.2014.5641 PMC4698984

[33] Jędzierowska, M. & Koprowski, R. Novel dynamic corneal response parameters in a practice use: a critical review. *Biomed. Eng. Online* **18**, 17 (2019). 30760270 10.1186/s12938-019-0636-3 PMC6375180

[34] Zhang, P. et al. CorNet: Autonomous feature learning in raw Corvis ST data for keratoconus diagnosis via residual CNN approach. *Comput. Biol. Med.* **172**, 108286 (2024). 38493602 10.1016/j.compbiomed.2024.108286

[35] Wang, B., Yang, L., Cheng, J., Wang, J. & Mei, Y. In-vivo high-speed biomechanical imaging of the cornea using Corvis ST and digital image correlation. *Comput. Biol. Med.* **153**, 106540 (2023). 36646022 10.1016/j.compbiomed.2023.106540

[36] Liu, T. et al. Changes and quantitative characterization of hyper-viscoelastic biomechanical properties for young corneal stroma after standard corneal cross-linking treatment with different ultraviolet-A energies. *Acta Biomater* **113**, 438–451 (2020). 32525050 10.1016/j.actbio.2020.06.005

[37] Liu, T. et al. Characterization of hyperelastic mechanical properties for youth corneal anterior central stroma based on collagen fibril crimping constitutive model. *J. Mech. Behav. Biomed. Mater.* **103**, 103575 (2020). 32090903 10.1016/j.jmbbm.2019.103575

[38] Li, H. et al. Biomechanical effect of ultraviolet-A-riboflavin cross-linking on simulated human corneal stroma model and its correlation with changes in corneal stromal microstructure. *Exp. Eye Res.* **197**, 108109 (2020). 32565111 10.1016/j.exer.2020.108109

[39] Mazinani, P., Cardillo, C. & Mosaddegh, P. Evaluating corneal biomechanics using shear wave elastography and finite element modeling: sensitivity analysis and parametric optimization. *Continuum Mech. Thermodyn.* **37**, 12 (2025).

[40] Mazinani, P. & Terranova, L. M. Finite element simulation for finding shear wave velocity on the canine cornea and sensitivity analysis for intraocular pressure parameter. *Mech. Res. Commun.* **150**, 104558 (2025).

[41] Mazinani, P., Terranova, L. M. & Setayeshnasab, H. Evaluating corneal biomechanics using intraocular pressure methods and finite element modeling: parameters study and parametric optimization. *Z. Angew. Math. Phys.* **76**, 220 (2025).

[42] Qin, X. et al. Determine corneal biomechanical parameters by finite element simulation and parametric analysis based on ORA measurements. *Front. Bioeng. Biotechnol.* **10**, 862947 (2022). 35497338 10.3389/fbioe.2022.862947 PMC9043460

[43] Mazinani, P. & Cardillo, C. Shear wave velocity and finite element modeling for understanding keratoconus biomechanics: comparison with healthy cornea. *Math. Mech. Solids* (2025).

